# The clinical and pathological characteristics of IgA nephropathy patients in Tibet

**DOI:** 10.1186/s12882-022-02895-4

**Published:** 2022-07-27

**Authors:** Fenglei Si, Jiarong Mei, Yong A, Chen Tang, Yuxuan Yao, Lijun Liu

**Affiliations:** 1 Renal Division, Peking University First Hospital, Peking University Institute of Nephrology, Beijing, China; 2 Key Laboratory of Renal Disease, Ministry of Health of China, Key Laboratory of Chronic Kidney Disease Prevention and Treatment (Peking University), Ministry of Education, Beijing, China; 3 Research Units of Diagnosis and Treatment of Immune-Mediated Kidney Diseases, Chinese Academy of Medical Sciences, Xishiku Street No.8, 100034 Beijing, China; 4 Eight-Year-Program, Grade 2017, Health Science Center, Peking University, Beijing, China; 5 Renal Division, People’s Hospital of Tibet Autonomous Region, Lasa, China

**Keywords:** Tibet, IgA nephropathy, Clinical, Pathological

## Abstract

**Background:**

There are few studies on immunoglobulin A nephropathy (IgAN) at high altitude. This study aimed to analyze the clinical and pathological characteristics of IgAN between Tibet and Beijing, which provided a basis for improving diagnosis and treatment in Tibet.

**Method:**

The clinical and pathological data of 80 patients from the People’s Hospital of Tibet Autonomous Region (Tibetan group) and 991 patients from Peking University First Hospital (Beijing group) with IgAN proven by renal biopsy were compared retrospectively between January 2016 and July 2020. The kidney biopsy tissue was sent to the Department of Nephrology, Peking University First Hospital for pathological evaluation.

**Results:**

The proteinuria (2.9 [2.0, 4.9] vs. 1.1 [0.5, 2.4] g/day, *P* < 0.001) in the Tibetan group was significantly higher than that in the Beijing group. The serum albumin (30.4 ± 7.7 vs. 38.2 ± 5.5 g/L, *P* < 0.001) was significantly lower in the Tibetan group. The eGFR (77.7 ± 37.8 vs. 62.1 ± 33.6 ml/min/1.73 m^2^, *P* = 0.001) was higher in the Tibetan group. The percentage of patients with nephrotic syndrome in the Tibetan group was significantly higher than that in the Beijing group (33.8% vs. 4.7%, *P* < 0.001).

**Conclusion:**

There are differences in the clinical and pathological characteristics of IgAN between plateau and plain regions.

## Background

Immunoglobulin A (IgA) nephropathy (IgAN) is the most prevalent primary chronic glomerular disease worldwide [1]. There are numerous studies about IgAN; however, relevant reports in high-altitude regions are few. Due to the unique geographical features and atmospheric conditions at high altitudes, such as deficient oxygen pressure, dry climate and strong ultraviolet radiation, the clinical and pathological characteristics of IgAN patients living in these regions may differ from those living in plain areas [2, 3]. Our study mainly retrospectively compared and analyzed the clinical and pathological characteristics of patients with IgAN between Tibet and Beijing to provide a basis for improving diagnosis and treatment in Tibet.

## Method

### Patients

The People’s Hospital of Tibet Autonomous Region in Tibet (Tibetan group) and Peking University First Hospital in Beijing (Beijing group) were chosen as the comparative hospitals. This study included 80 patients in the People’s Hospital of Tibet Autonomous Region and 991 patients in Peking University First Hospital, who were all diagnosed with primary IgA nephropathy by renal biopsy from January 2016 to July 2020. Secondary IgAN, such as chronic liver disease, Henoch-Schönlein Purpura or concomitant connective tissue disease was excluded. We also excluded IgAN with minimal renal disease changes or focal segmental glomerulosclerosis by comparison of light, immunofluorescence and in 60% of cases, electron microscopy. Kidney tissue from the Tibetan group was sent to the Department of Nephrology, Peking University First Hospital for pathological evaluation. They were examined by light microscopy, immunofluorescence and electron microscopy by two pathologists.

### Data collection

All of the above patients had complete clinical records and pathological data. The data used for analysis included sex, age, systolic and diastolic blood pressure (SBP and DBP, respectively), hemoglobin (HGB), platelets (PLT), serum albumin (ALB), 24-h urine total protein (UTP), serum creatinine (Scr), serum uric acid (UA), serum phosphate (P), albumin-corrected serum calcium (Ca_Alb_), estimated glomerular filtration rate (eGFR), serum IgG, serum IgA, serum IgM, serum C3 and serum C4 at the time of renal biopsy. Mean arterial pressure (MAP) was calculated as DBP plus one-third of the pulse. The eGFR was calculated by using the Chronic Kidney Disease Epidemiology Collaboration (CKD-EPI) creatinine equation [4].

The renal biopsies were scored according to the updated Oxford classification of IgA nephropathy (MEST-C scoring system) proposed by the IgA Nephropathy Classification Working Group; however, there were no C classifications in the early cases [5]. We also collected immune deposits IgA, IgM, IgG, C3 and C1q of renal biopsy samples by immunofluorescence technique. The intensity was determined semi-quantitatively using a scale from 0 to 4 + .

### Statistical analysis

Normally distributed data are presented as the means ± SDs, and nonnormally distributed data are presented as the medians [Q25, Q75]. Categorical data are summarized as counts and percentages. Clinical parameters are compared using Student’s t-tests (for normally distributed, continuous variables), Wilcoxon signed-rank tests (for nonnormally distributed, continuous variables) or χ2 tests (for nominal variables), as appropriate. All analyses were performed using SPSS Statistics, version 26.0 (SPSS Inc., Chicago, IL, USA). A *p* value less than 0.05 was considered statistically significant.

## Results

### Demographic and clinical data

Table 1 summarizes the characteristics of the patients in the Tibetan group and Beijing group. The patients in the Tibetan group were significantly younger than those in the Beijing group (32.6 ± 12.4 vs. 39.12 ± 12.6 years, *P* < 0.001). However, there was no difference in sex between the two groups (*P* > 0.05). The mean arterial pressure (106.0 ± 19.0 vs. 97.0 ± 12.6 mmHg, *P* < 0.001), hemoglobin (158.6 ± 83.8 vs. 126.3 ± 20.9 g/L, *P* < 0.001), proteinuria (2.9 [2.0, 4.9] vs. 1.1 [0.5, 2.4] g/day, *P* < 0.001), eGFR (77.7 ± 37.8 vs. 62.1 ± 33.6 ml/min/1.73 m^2^, *P* = 0.001), serum phosphate (1.30 ± 0.29 vs. 1.17 ± 0.27 mmol/L, *P* < 0.001), serum IgM (1.14 [0.92, 1.53] vs. 0.94 [0.68, 1.33] g/L, *P* = 0.003), serum C3 (1.18 [1.04, 1.47] vs. 0.85 [0.74, 0.98] g/L, *P* < 0.001) and the C3/C4 ratio (4.44 [3.84, 5.61] vs. 3.60 [3.06, 4.15], *P* < 0.001) in the Tibetan group were significantly higher than those in the Beijing group. The serum albumin (30.4 ± 7.7 vs. 38.2 ± 5.5 g/L, *P* < 0.001), albumin-corrected serum calcium (2.28 ± 0.33 vs. 2.33 ± 0.17 mmol/L, *P* = 0.017), serum IgG (8.66 [5.41,12.62] vs. 10.30 [8.49,12.50] g/L, *P* = 0.025) and the IgA/C3 ratio (2.52 [1.89, 3.16] vs. 3.58 [2.74, 4.63], *P* < 0.001) were significantly lower in the Tibetan group. The percentage of patients with nephrotic syndrome in the Tibetan group was significantly higher than that in the Beijing group (33.8% vs. 4.7%, *P* < 0.001). However, there were no differences in platelets, uric acid, serum IgA or C4 in the two groups (*P* > 0.05).

**Table 1:** Comparison of clinical characteristics between the two groups at the time of renal biopsy

	Tibetan group (*n* = 80)	Beijing group (*n* = 991)	*P* Value
Ethnicity (Han/Tibetan)	1/79	990/1	< 0.001
Gender (M/F)	48/32	527/464	0.239
Age (years)	32.6 ± 12.4	39.12 ± 12.6	< 0.001
SBP (mmHg)	135.7 ± 24.6	129.3 ± 16.3	0.043
DBP (mmHg)	91.2 ± 17.9	80.9 ± 11.7	< 0.001
MAP (mmHg)	106.0 ± 19.0	97.0 ± 12.6	< 0.001
HGB (g/L)	158.6 ± 83.8	126.3 ± 20.9	< 0.001
PLT (10^9/L)	239.8 ± 89.7	239.2 ± 68.5	0.77
ALB (g/L)	30.4 ± 7.7	38.2 ± 5.5	< 0.001
UTP (g/day)	2.9 [2.0, 4.9]	1.1 [0.5, 2.4]	< 0.001
Nephrotic syndrome	27/80 (33.8%)	47/991 (4.7%)	< 0.001
eGFR (ml/min/1.73 m^2^)	77.7 ± 37.8	62.1 ± 33.6	0.001
UA (μmol/L)	424.1 ± 109.1	414.4 ± 115.2	0.401
Ca_Alb_ (mmol/L)	2.28 ± 0.33	2.33 ± 0.17	0.017
P (mmol/L)	1.30 ± 0.29	1.17 ± 0.27	< 0.001
Serum IgG (g/L)	8.66 [5.41, 12.62]	10.30 [8.49, 12.50]	0.025
Serum IgA (g/L)	2.80 [2.29, 3.92]	3.13 [2.38, 3.91]	0.54
Serum IgM (g/L)	1.14 [0.92, 1.53]	0.94 [0.68, 1.33]	0.003
Serum C3 (g/L)	1.18 [1.04, 1.47]	0.85 [0.74, 0.98]	< 0.001
Serum C4 (g/L)	0.27 [0.21, 0.34]	0.24 [0.20, 0.29]	0.066
Serum C3/C4	4.44 [3.84, 5.61]	3.60 [3.06, 4.15]	< 0.001
Serum IgA/C3	2.52 [1.89, 3.16]	3.58 [2.74, 4.63]	< 0.001

*Abbreviations*: *SBP* Systolic blood pressure, *DBP* Diastolic blood pressure, *MAP* Mean arterial pressure, *HGB* Hemoglobin, *PLT* Platelets, *ALB* Serum albumin, *UTP* 24-h urine total protein, *Scr* Serum creatinine, *eGFR* Estimated glomerular filtration rate (calculated using the CKD-EPI equation), *UA* Serum uric acid, *Ca*_*Alb*_ Albumin-corrected serum calcium_,_ *P* Serum phosphate

### Clinicopathological features of the renal biopsy samples

As shown in Table 2, for the renal pathological scores according to the new Oxford classification there were no differences between the two groups [5]. The IgA deposits (2.5 [2.0, 3.0] vs. 3.0 [3.0, 3.5], *P* < 0.001) and C3 deposits (1.0 [0.5, 2.0] vs. 2.5 [2.0, 3.0], *P* < 0.001) in kidney tissue were less in Tibetan group than those of Beijing group. There were no differences in IgG, IgM, C1q deposits between the two groups.

**Table 2:** Comparison of pathological features between the two groups of patients^a^

	Tibetan group (*n* = 70)	Beijing group (*n* = 977)	*P* Value
M0/M1	19/51	345/632	0.166
E0/E1	36/34	597/380	0.11
S0/S1	26/44	291/686	0.196
T0/T1/T2	43/20/7	489/356/132	0.183
C0/C1/C2^b^	24/34/9	257/466/70	0.312
IgA deposits	2.5 [2.0, 3.0]	3.0 [3.0, 3.5]	< 0.001
IgG deposits	0.0 [0.0, 0.0]	0.0 [0.0, 0.0]	0.087
IgM deposits	1.0 [0.0, 1.1]	0.5 [0.0, 1.0]	0.613
C3 deposits	1.0 [0.5, 2.0]	2.5 [2.0, 3.0]	< 0.001
C1q deposits	0.0 [0.0, 0.0]	0.0 [0.0, 0.0]	0.779

*M* Mesangial hypercellularity, *M0* < 50% of glomeruli showing mesangial hypercellularity, *M1* > 50% of glomeruli showing mesangial hypercellularity, *E* Endocapillary hypercellularity, *E0* No endocapillary hypercellularity, *E1* Any glomeruli showing endocapillary hypercellularity, *S* Segmental glomerulosclerosis, *S0* absent, *S1* Present in any glomeruli, *T* Tubular atrophy/interstitial fibrosis, *T0* 0–25% of cortical area, *T1* 26–50% of cortical area, *T2* > 50% of cortical area, *C* Cellular or fibro-cellular crescents, *C0* absent, *C1* 0–25% of glomeruli, *C2* ≥ 25% of glomeruli

^a^Some renal biopsy samples could not be scored by the MEST-C scoring system due to few glomeruli

^b^There were no C classification in the early cases. Sixty-seven patients in the Tibetan group and 793 patients in the Beijing group had C scores

## Discussion

In Tibet, due to limited renal biopsy technology, pathological classification data for chronic kidney disease patients are extremely limited, which obviously affects the clinical diagnosis, design of therapeutic regimens and prognostic evaluation [6]. Since the medical groups aided in Tibet in 2015, with the help of experienced nephrologists from Peking University First Hospital, the local hospital has successfully carried out more kidney biopsies, which enabled more IgAN patients to be diagnosed.

Our study examined data regarding the clinical and pathological characteristics of IgAN from the plateau regions from January 2016 to July 2020. There have been few previous studies of kidney biopsy data about IgAN from plateau regions, and this may be the first report with the largest number of cases of IgAN in these regions. We found that there were differences in renal clinical features between IgAN patients in plateau and plain regions.

One valuable finding in our study is that the amount of urine protein in the Tibetan group was significantly higher than that in the Beijing group, which was consistent with previous studies [7, 8]. The pathogenesis of the proteinuria at high altitude may relate to a variety of factors, including the effects of tissue hypoxia within the kidney parenchyma, glomerular capillary hypertension, hyperviscosity and elevated right heart pressures [7, 8].

It is well known that nephrotic syndrome in IgAN is an uncommon manifestation, except in patients with minimal-change disease [9]. The prevalence of 14.7% NS in IgAN in a Chinese cohort was reported by Han et al [10]. In a Korean cohort, 10.2% of IgAN patients were found to present NS [11]. However, the incidence of nephrotic syndrome in the Tibetan group was 33.8%, which was significantly higher. The above clinical data suggest that the clinical manifestations of IgAN patients in Tibet are significantly heavier than those in the Beijing group. In addition to the relatively late visit time, it may also be related to the high-altitude environment, ethnicity, hyperuricemia, high-altitude hypertension and high infection rate [12, 13].

Regarding the secondary clinical features between the two groups in our study, the blood pressure of IgAN patients in the Tibetan group was higher than that in the Beijing group. This may be related to high-sodium diets and the high altitude of the Tibetan Plateau [14, 15], which may be one of the reasons for the increasement in proteinuria and eGFR in Tibetan patients [16]. In addition, the higher eGFR in the Tibetan group might be related to the younger ages in the Tibetan group. However, due to the higher blood pressure and urinary protein, the eGFR might decline rapidly in the Tibetan group in the future. Regrettably, our study lacked a prognostic data. In the Tibetan group, the hemoglobin was also higher, which may be related to the secondary polycythemia caused by high altitude and hypoxia in the Tibetan Plateau [17, 18].

Another major finding in the present study was that the serum C3 levels and the C3/C4 ratio were significantly higher and the IgA/C3 ratio was lower in the Tibetan group than those in the Beijing group [19–21]. Meanwhile, we also found that IgA and C3 deposits in kidney tissue were less in Tibetan group than those in Beijing group. Previous studies showed that glomerular complement activation was strongly associated with IgAN and may be the dominant driver of renal injury [22–24]. Therefore, our findings suggested there might be less complement activation in the Tibetan group than that in the Beijing group, which may at least in part explain the relatively better chronic kidney lesions and renal function observed in patients in the Tibetan group compared with those of the Beijing group. However, the detailed differences of glomerular complement activation between two groups remain to be further explored.

There are several limitations in our study. First, this was a single-center retrospective study with a lack of detailed follow-up and prognostic data. Second, our sample size was relatively small. Third, we also lacked studies about the mechanisms of IgAN in Tibet. Nevertheless, to our knowledge, this was the first study with the largest sample size to describe IgAN in Tibet.

## Conclusions

In conclusion, there are differences in the clinical and pathological characteristics of IgAN between plateau and plain regions. Despite limitations in this study, it may provide insight into IgAN patients’ clinical features in plateau regions.

## Data Availability

The datasets used and/or analysed during the current study are available from the corresponding author upon reasonable request.

## Abbreviations

IgAN: Immunoglobulin A nephropathy
SBP: Systolic blood pressure
DBP: Diastolic blood pressure
MAP: Mean arterial pressure
HGB: Hemoglobin
PLT: Platelets
ALB: Serum albumin
UTP: 24-H urine total protein
Scr: Serum creatinine
eGFR: Estimated glomerular filtration rate (calculated using the CKD-EPI equation)
UA: Serum uric acid
Ca_Alb_: Albumin-corrected serum calcium
P: Serum phosphate
